# Socio-demographic and Clinical Predictors of Relapses in Severe Mental Disorders: A Cross-sectional Study at El Razi Hospital, Tunisia

**DOI:** 10.1192/j.eurpsy.2026.12190

**Published:** 2026-07-31

**Authors:** S. Ben Othmen, M. Sehli, M. A. Yaiche, H. Mami, A. Aissa

**Affiliations:** Taher Maamouri Hospital, Nabeul, Tunisia

## Abstract

**Introduction:**

Relapses in schizophrenia and bipolar disorder are common and contribute to poor functional outcomes and frequent hospitalizations. Understanding the factors associated with relapse is essential to improve long-term management. In Tunisia, where psychiatric resources are limited, such data remain scarce and context-specific evidence is needed.

**Objectives:**

This study aimed to identify socio-demographic and clinical predictors of relapse in a Tunisian cohort.

**Methods:**

A descriptive cross-sectional study was conducted including 87 hospitalized patients (43 BD, 44 SCZ) aged 18–55 years, from March to June 2022. Socio-demographic data, personal and family psychiatric history, and validated scales (FACES IV, DAI-10, Birchwood Insight Scale, IEV) were collected. Analyses were performed using SPSS 22.0 (Chi-square, Kruskal–Wallis).

**Results:**

Mean age was 37.3±9.9 years; male-to-female ratio 2:1. Most patients were single (70%), had primary education (45%), and were unemployed (64%); 50% lacked health coverage. Mean age at first hospitalization was 26.5±7.0 years; untreated psychosis in SCZ averaged 24 months. Annual relapse rate was 0.96. Significant predictors of relapse included age <35 (p<0.001), single/divorced status (p=0.012), substance use (78% smokers, 34% alcohol, 26% cannabis; p=0.007), DPNT >12 months (p=0.036), absence of consanguinity (protective; p=0.014), and poor inter-episodic interval quality (p=0.017).

**Conclusions:**

Young age, single/divorced status, substance use, and prolonged untreated psychosis are major predictors of relapse. These findings highlight the need for early intervention, substance-use prevention, and targeted support for high-risk patients.

**Disclosure of Interest:**

None Declared

